# Decreased calcium permeability caused by biallelic *TRPV5* mutation leads to autosomal recessive renal calcium-wasting hypercalciuria

**DOI:** 10.1038/s41431-024-01589-9

**Published:** 2024-03-25

**Authors:** Naz Guleray Lafci, Mark van Goor, Semra Cetinkaya, Jenny van der Wijst, Melisa Acun, Fatma Kurt Colak, Arda Cetinkaya, Joost Hoenderop

**Affiliations:** 1 Hacettepe University, Medical Faculty, Department of Medical Genetics, Ankara, Turkey; 2 Health Science University, Dr. Sami Ulus Obstetrics and Gynecology, Children Health and Disease Training and Research Hospital, Department of Medical Genetics, Ankara, Turkey; 3 Department of Medical Biosciences, Radboud University Medical Center, Nijmegen, The Netherlands; 4 Health Science University, Dr. Sami Ulus Obstetrics and Gynecology, Children Health and Disease Training and Research Hospital, Department of Pediatric Endocrinology, Ankara, Turkey; 5 Hacettepe University, Institute of Health Sciences, Department of Bioinformatics, Ankara, Turkey

**Keywords:** Genetics research, Acid, base, fluid, electrolyte disorders

## Abstract

Hypercalciuria is the most common metabolic risk factor in people with kidney stone disease. Its etiology is mostly multifactorial, although monogenetic causes of hypercalciuria have also been described. Despite the increased availability of genetic diagnostic tests, the vast majority of individuals with familial hypercalciuria remain unsolved. In this study, we investigated a consanguineous pedigree with idiopathic hypercalciuria. The proband additionally exhibited severe skeletal deformities and hyperparathyroidism. Whole-exome sequencing of the proband revealed a homozygous ultra-rare variant in *TRPV5* (NM_019841.7:c.1792G>A; p.(Val598Met)), which encodes for a renal Ca^2+^-selective ion channel. The variant segregates with the three individuals with hypercalciuria. The skeletal phenotype unique to the proband was due to an additional pathogenic somatic mutation in *GNAS* (NM_000516.7:c.601C>T; p.(Arg201Cys)), which leads to polyostotic fibrous dysplasia. The variant in *TRPV5* is located in the TRP helix, a characteristic amphipathic helix that is indispensable for the gating movements of TRP channels. Biochemical characterization of the TRPV5 p.(Val598Met) channel revealed a complete loss of Ca^2+^ transport capability. This defect is caused by reduced expression of the mutant channel, due to misfolding and preferential targeting to the proteasome for degradation. Based on these findings, we conclude that biallelic loss of *TRPV5* function causes a novel form of monogenic autosomal recessive hypercalciuria, which we name renal Ca^2+^-wasting hypercalciuria (RCWH). The recessive inheritance pattern explains the rarity of RCWH and underscores the potential prevalence of RCWH in highly consanguineous populations, emphasizing the importance of exploration of this disorder within such communities.

## Introduction

Hypercalciuria is a condition of increased urinary calcium (Ca^2+^) excretion, present in ~4-10% of the otherwise healthy population [1, 2]. It is defined as >250 mg/24 h urine and >200 mg/24 h urine in men and women respectively, and an increased urinary calcium/creatinine ratio compared to age-matched children [3, 4]. People who suffer from hypercalciuria are mostly asymptomatic but may have nephrocalcinosis or increased bone resorption [5, 6]. Hypercalciuria is multifactorial, with an estimated heritability of ~50% [7]. To date, at least 25 genes have been linked to monogenic hypercalciuria, some accompanied by other systemic findings [8]. Nevertheless, the basis of hypercalciuria is still unidentifiable in the majority [8]. Many of these people have idiopathic hypercalciuria (IH), which is used as an umbrella term for hypercalciuric individuals with normocalcemia and no other systemic diseases, such as hyperparathyroidism [9].

Hypercalciuria can arise due to defects in three organs/systems and is classified according to the primary defect as absorptive (intestine), resorptive (bone), or renal leak type hypercalciuria [10]. These three organs collaborate to maintain a steady blood Ca^2+^ concentration and the interplay between them is controlled by a hormonal feedback loop mainly involving parathyroid hormone (PTH) and 1,25-dihydroxy vitamin D_3_ (calcitriol) [8]. The main role of kidneys is to regulate Ca^2+^ excretion. The fine-tuning of renal Ca^2+^ reabsorption takes place in the distal convoluted (DCT) and connecting tubules (CNT) [8]. TRPV5 (formerly known as ECaC1, Epithelial Calcium Channel 1), encoded by the *TRPV5* gene on 7q34 [MIM: 606679], is the main Ca^2+^ channel in the apical membranes. TRPV5-dependent Ca^2+^ transport constitutes the rate-limiting step in DCT/CNT-mediated Ca^2+^ reabsorption [11]. Knockout *Trpv5* mouse models, which suffer from a phenotype of renal Ca^2+^ wasting, illustrate the importance of this channel in Ca^2+^ homeostasis [12]. In addition, several human *TRPV5* variants have been associated with hypercalciuria and nephrolithiasis in case-control studies [13, 14]. Despite its role in Ca^2+^ homeostasis, *TRPV5* is yet to be linked to a monogenic disorder [11].

In this study, we explored the genetic cause of hypercalciuria driven by renal Ca^2+^ wasting in a family of consanguineous parents and three affected individuals. We employed a combination of homozygosity mapping and massively parallel sequencing to identify a homozygous missense mutation in *TRPV5* in all affected individuals. The proband also demonstrated hyperparathyroidism and severe bone deformities explained by a blended phenotype due to an additional somatic *GNAS* mutation. Biochemical and functional analyses on the mutated TRPV5 channel demonstrated failure to increase plasma membrane Ca^2+^ permeability in HEK293 cells and increased proteasomal degradation. Taken together, our data demonstrate that biallelic *TRPV5* mutations are responsible for a novel form of monogenic hypercalciuria in humans, which we term renal calcium-wasting hypercalciuria (RCWH).

## Subjects and methods

### Study participants

A single-family of 8 individuals was evaluated for hypercalciuria by urinary Ca^2+^/creatinine ratio (uCa/Cr) from either spot or 24-hour urine samples. uCa/Cr levels were compared to age-matched reference values [4]. Additionally, all individuals underwent physical examination and systematic evaluation. Genomic DNA was extracted using QIAamp DNA Mini Kit (Qiagen, Hilden, Germany) from the peripheral blood of all family members. Additionally, peripheral whole blood RNA was obtained from the proband (II-2) for splice-site sequencing (Supplementary Information).

The study protocol was approved by the local ethics committees of Hacettepe University and Dr. Sami Ulus Obstetrics and Gynecology, Children Health and Disease Training and Research Hospital (GO21/446, 30.03.2021, and 2012-KAEK-15/2030, 22.01.2020). The study was conducted in accordance with the Declaration of Helsinki and written informed consent was obtained from all participants and/or their parents.

### DNA sequencing

Whole-exome sequencing (WES) using peripheral blood DNA from the proband was utilized to uncover the genetic etiology. WES library was generated using Twist Human Core Exome Kit v2 (Twist Bioscience, San Francisco, CA, USA) and was sequenced on the Illumina NextSeq 500 (Illumina, San Diego, CA, USA) platform. Sequence reads were aligned to hg19/GRCh37 and germline/somatic variants were called and analyzed following the pipelines provided in Supplementary Information. The strong implication of identity-by-descent prompted us to perform homozygosity mapping for all family members and focus variant analysis on shared long contiguous stretches of homozygosity (LCSHs) in either all individuals with hypercalciuria (II:1, II:2, II:6) or those unique to the proband (II:2). Furthermore, gene panels were adopted into the pipeline as detailed in Supplementary Information.

Any candidate variants were inspected via Integrative Genomics Viewer (IGV) and sequenced in family members by Sanger sequencing using BigDye Terminator v3.1 (ThermoFisher Scientific, Waltham, MA, USA) on ABI 3500 Genetic Analyzer (ThermoFisher Scientific, Waltham, MA, USA) for evaluating segregation and validation.

### Homozygosity mapping

For all eight individuals, genome-wide single nucleotide polymorphism (SNP) genotyping was performed using Infinium HumanCytoSNP-12 v2.1 microarrays, according to the manufacturer’s protocol. GenomeStudio software v2.0 (Illumina, San Diego, CA, USA) was utilized for calling SNP genotypes according to hg19/GRCh37, and obtained genotypes were viewed by MS-Excel to look for ≥2 Mbp LCSHs, indicating homozygosity-by-descent.

### Generation of TRPV5 p.(Val598Met) construct

Site-directed mutagenesis was used to introduce a point mutation in the wildtype *TRPV5* sequence, using the Q5 hot-start kit (New England Biolabs, Ipswich, MA, USA) according to the manufacturer’s protocol. Details of the protocol are presented in Supplementary Information. The resulting construct contained the *TRPV5* p.(Val598Met) ORF, CMV promoter, and GFP tag and was validated using Sanger sequencing.

### Functional evaluation of TRPV5 in HEK293 cells

HEK293 cells were transfected with the aforementioned constructs using Lipofectamine2000 (Thermo Fisher Scientific, Waltham, MA, USA). TRPV5 protein levels were evaluated in a semi-quantitative manner using Western Blotting, either on total cellular proteins or on cell-surface proteins marked by biotinylation. The effect of the mutation on protein stability was assessed using inhibitors of proteasomal (MG-132 (MG), 5 µM, obtained from Merck, Darmstadt, Germany) and lysosomal (Bafilomycin A1 (Baflo), 100 nM, obtained from Cell Signaling Technology, Beverly, MA, USA) degradation. Finally, the permeability of the TRPV5 channel was evaluated using a previously described radioactive ^45^Ca^2+^ uptake protocol [15]. Each assay and statistical method are detailed in Supplementary Information.

## Results

### Clinical features of the affected individuals

**Fig. 1:**
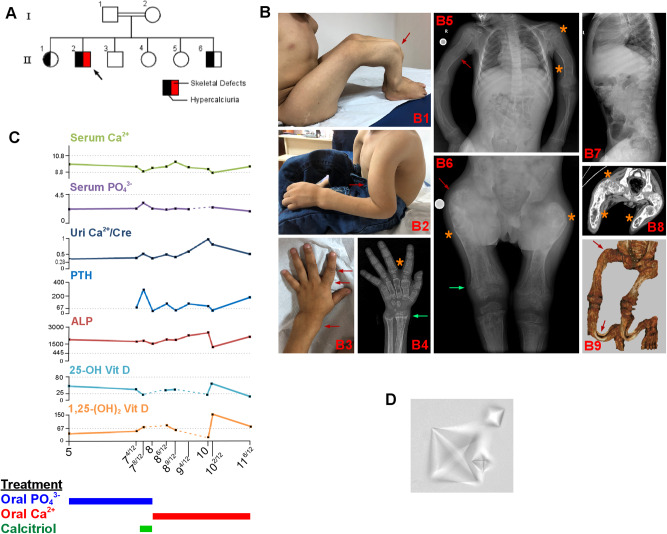
Clinical findings of the proband. **A** The hypercalciuria and skeletal defects running in the family are indicated on the pedigree. **B** Photographs and radiological images of the proband demonstrate severe skeletal deformities. Radiographs revealed generalized osteopenia. The severe bowing deformities in the right leg (B1, red arrow) and left arm (B2, red arrow) are seen upon inspection, along with the widening of the left wrist, metacarpophalangeal and interphalangeal joints (B3, red arrows). The bone age is delayed and appropriate for ~4 years (B4). Radiographs (B4-6) reveal extensive POFD-related findings; including the characteristic “ground-glass” appearance; composed of mixed radiolucent (cystic) and sclerotic lesions indicated by orange asterisks, metaphyseal flaring & cupping (green arrows), and bowing of long bones (red arrows). Occasional cortical thinning and medullary enlargement of the long bones are also evident. Vertebrae appear spared (B7). The computed tomography scans also show radiolucent bony lesions with sclerotic rims (B8, orange asterisks) and 3D-reconstructions of CT scans demonstrate bowing in femora (Shepherd’s crook sign) and tibiae (red arrows). Bowing of the bones within radiological images indicates poorly healed new and old appendicular fractures. **C** Age-dependent change in Ca^2+^ metabolism-related biochemical parameters of the proband is shown. Any missing measurements are indicated as dashed lines. The three rectangles at the bottom indicate the various treatments the proband received over time. **D** Urinalysis reveals multiple typical octahedral calcium oxalate crystals in the proband.

The 10-year old proband has been evaluated at the Pediatric Endocrinology and Medical Genetics Departments of Dr. Sami Ulus Obstetrics and Gynecology, Children Health and Disease Training and Research Hospital for short stature, bone deformities with several fractures, and hypercalciuria. He was the second of six siblings born to consanguineous parents of Syrian origin (Fig. 1A). He appeared unaffected at birth and started to walk independently when he was 1 year old. At 1.5 years, parents noticed unusual bending of the limbs, gradually progressing and becoming painful. During his first visit to the medical genetics clinic at 7 years old, he was not able to walk. He had caput quadratum, thickening of joints, and bent limbs, also documented by radiological imaging (Fig. 1B). Neurodevelopmental milestones throughout his life and cognitive development assessed at 7.5 years were clinically appropriate for his age. Ophthalmologic, audiologic, and dermatologic evaluations were normal, and echocardiography revealed a patent foramen ovale.

The combination of the proband’s skeletal findings and hypercalciuria resembled severe rickets or osteogenesis imperfecta. At 5 years, laboratory tests to evaluate Ca^2+^ metabolism revealed hypercalciuria, normocalcemia, hypophosphatemia, hyperphosphaturia, elevated serum alkaline phosphatase (ALP) and parathyroid hormone (PTH) but with normal 25-hydroxy vitamin D_3_ and calcitriol levels (Fig. 1C and Supplementary Table S1). Additionally, the Tc-99m sestamibi scintigraphy in the proband did not reveal any abnormal uptake by parathyroids. This metabolic profile partially coincided with those seen in four disorders; (1) primary hyperparathyroidism, which contradicts proband’s normocalcemia and normal parathyroid scintigraphy; (2) hereditary hypophosphatemic rickets except for proband’s hypercalciuria; (3) hereditary hypophosphatemic rickets with hypercalciuria (HHRH) except for hyperparathyroidism and normal calcitriol levels seen in the proband; (4) Polyostotic fibrous dysplasia (POFD) albeit hyperparathyroidism and hypercalciuria. Although each of these conditions differed from the proband’s metabolic profile to some extent, initially sodium phosphate was administered for HHRH treatment. Additionally, due to low 1,25-(OH)_2_ Vitamin D levels, calcitriol was supplemented, which did not benefit the calcium metabolism and was eventually discontinued. After the identification of a potentially pathogenic variant in *TRPV5*, the treatment was tailored towards a renal Ca^2+^-wasting condition. Prior treatments were replaced by oral Ca^2+^-lactate, which succeeded in decreasing PTH levels but failed to improve bone phenotype in the short term. The bone turnover parameters under this treatment indicated increased bone formation, represented by continuously high ALP and osteocalcin levels with normal bone resorption characterized by normal urinary deoxypyridinoline excretion. The evolution of Ca^2+^ metabolism indicators is summarized in Fig. 1C and details can be found in Supplementary Table S1.

Repeated renal ultrasounds during the proband’s follow-up revealed intermittent kidney stones and microscopic urinalysis identified multiple calcium oxalate crystals (Fig. 1D). He consistently had normal urinary pH, and no glucosuria, aminoaciduria, or proteinuria. His siblings were also screened for hypercalciuria (Supplementary Table S1). These measurements demonstrated persistent hypercalciuria in individuals II-1, II-2, and II-6. None of the skeletal abnormalities found in the proband were observed in these individuals. The mother (individual I-2) had nephrolithiasis during her fifth pregnancy at 25 years of age, presumptively suggesting that heterozygosity for the *TRPV5* variant may be a predisposing factor for nephrolithiasis.

### Genome-wide search for the cause of hypercalciuria in the family identifies biallelic p.(Met598Val) in *TRPV5*

**Fig. 2:**
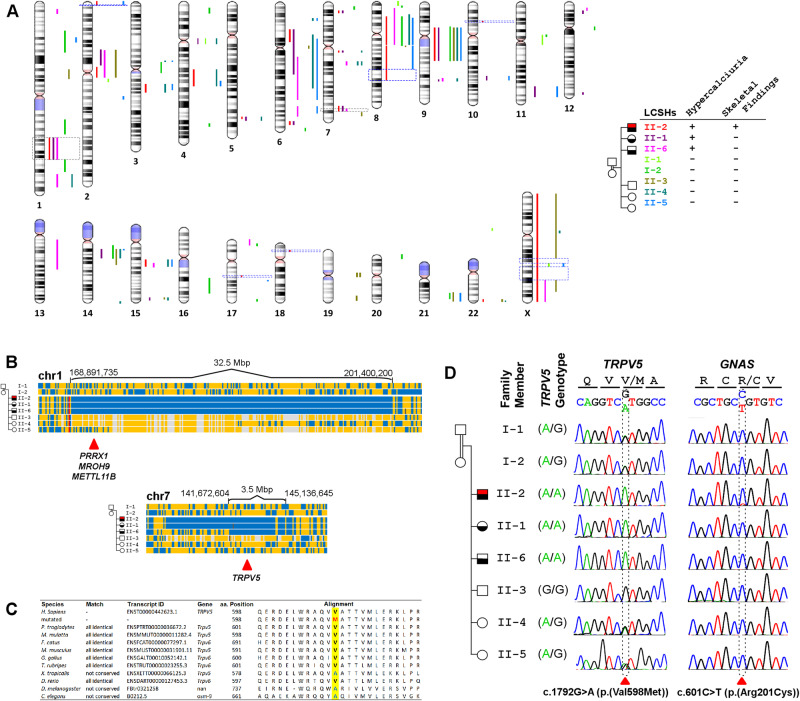
Investigation of the genetic disease etiology in the family. **A** LCSHs (≥2 Mbp) on all autosomes and the X chromosome are shown for each member of the family, specified by the color legend on the right. Gray dashed rectangles on chromosomes 1 (left) and 7 (right) indicated LCSHs shared by all affected individuals, while blue dashed rectangles on 7 genomic loci on 6 chromosomes correspond to LCSHs unique to the proband. **B** A detailed view of the two genomic loci common in all affected siblings is shown. Each row represents an individual genome and the vertical lines indicate the genotype for the aligned genomic loci. Homozygous and heterozygous loci in the proband (II-2) are indicated in blue and orange, respectively. The same color code is also valid for other individuals with the addition of white and red lines which symbolize contrasting homozygous genotypes and erroneous genotypes, respectively. Note that 4 genes with candidate variants on both chromosomes are marked with red arrowheads. **C** Evolutionary conservation of the 598^th^ residue of TRPV5 across 10 commonly used model organisms. Note that this position is occupied by either valine or alanine residues in all species, while the p.(Val598Met) variant replaces these with the bulkier methionine residue containing Sulfur. **D** Sanger electropherograms of the *TRPV5*:c.1792G>A and *GNAS*:c.601C>T variants reveal respective genotypes for all family members. Note that the somatic *GNAS* variant is only present in the proband with distinctively lower peak amplitude.

Considering the parental consanguinity and three hypercalciuric children, we carried out genome-wide homozygosity mapping of the whole family to explore genomic regions identical-by-descent. This revealed 2 shared LCSH regions in affected individuals (II-1, II-2, and II-6), which were located on 1q24.2–1q32.1 (168,891,735-201,400,200) and 7q33–7q35 (141,672,604–145,136,645) (Fig. 2A, B), excluding 98.85% of the genome. When high-quality (GQ ≥ 30 and read depth ≥20), homozygous, and rare (MAF ≤ 0.01 and no homozygous individual in gnomAD v4.0) variants in shared LCSHs from WES data were filtered in only 3 candidate variants remained (Table S2). The variant containing genes, *MROH9* and *METTL11B*, have no renal expression [16]. Additionally, these are unlikely to be disease-causing genes as healthy individuals harboring homozygous loss-of-function variants in *MROH9* have already been reported in gnomAD v4.0 and *METTL11B*-knockout mice have no significant phenotypic abnormality [17]. On the other hand, the only remaining candidate, *TRPV5*, is exclusively expressed in the kidney and under selection pressure in the healthy human population (LOEUF = 0.97) with no reported homozygous predicted loss-of-function variants in gnomAD v4.0 [16, 18]. The NM_019841.7:c.1792G>A (p.(Val598Met)) variant in the TRPV5 protein disturbed the highly conserved ‘VATVV’ motif (Fig. 2C). Sanger sequencing confirmed this variant in the proband and demonstrated cosegregation with hypercalciuria within the family (Fig. 2D).

Furthermore, the proband’s exceptionally severe skeletal phenotype hinted towards genetic variants besides *TRPV5* in this individual. For this purpose, we initially explored 7 LCSH regions unique to the proband (Fig. 2A). These regions contain 2 candidate high-quality, homozygous, and rare variants in *POP1* and *TSPAN6* which are excluded as shown in Table S2. Additionally, the synonymous variant 3 bp to the nearest intron-exon boundary in *POP1* did not alter the mRNA splicing (Supplementary Fig. S1). We also investigated the WES data with 2 virtual panels. The virtual Skeletal Disorders panel uncovered 8 candidate variants, which are further excluded (Table S2). On the other hand, the virtual Hypercalciuria/Hyper-PTH panel uncovered only the TRPV5 variant, but this variant being not unique to the proband, cannot explain his severe skeletal phenotype. Lastly, we looked for somatic variants in the virtual Skeletal Disorders panel and identified a well-characterized pathogenic *GNAS* variant (NM_000516.7:c.601C>T; p.(Arg201Cys)), exclusively present in the proband (Fig. 2D) with a variant allele fraction of 0.12 (Supplementary Fig. S2). In light of this finding, a reevaluation of the clinical and radiological findings established the diagnosis of POFD (OMIM 174800).

### TRPV5 p.(Val598Met) mutant channel is not functional

**Fig. 3:**
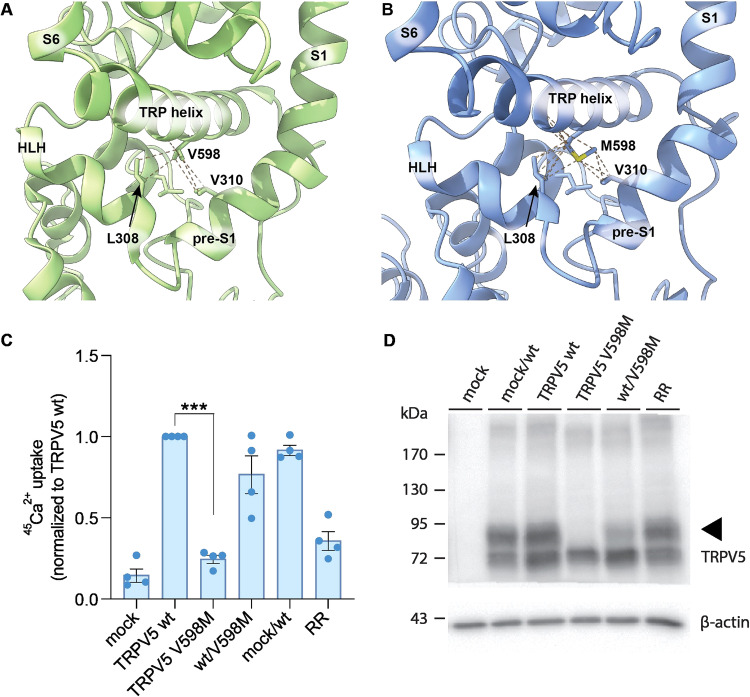
TRPV5 p.(Val598Met) function is impaired in ^45^Ca^2+^ uptake experiments. **A** Zoom of the wildtype TRPV5 channel structure (model obtained from the protein data bank (PDB) database with accession number: 6O1P) with p.(Val598Met) and its surroundings highlighted. Helix-loop-helix (HLH), transmembrane helices 1 and 6 (S1/S6), pre helix before first transmembrane helix (pre-S1). **B** Zoom of the mutated TRPV5 channel with a focus on the interactions that p.Met598 makes with residues in the pre-S1 and HLH domains. The mutated protein model was made by DynaMut2 prediction software. **C** Relative ^45^Ca^2+^ uptake in HEK293 cells transiently transfected with either mock, TRPV5 wildtype (wt), TRPV5 p.(Val598Met) (V598M), co-transfection of TRPV5 wildtype with TRPV5 p.Val598Met (wt/V598M), co-transfection of mock with TRPV5 wildtype (mock/wt) and transfection of TRPV5 wildtype treated with the TRPV5 blocker ruthenium red (RR). Uptake is normalized to the TRPV5 wt condition. The experiment consists of 4 independent biological replicates (*N* = 4) and individual data points corresponding to 3 technical replicates per condition. RR=ruthenium red. TRPV5 wt vs TRPV5 p.(Val598Met): *p* = 0.0026. **D** Representative immunoblot showing expression of TRPV5 (via HA-tag) and β-actin from one of the biological replicates of the ^45^Ca^2+^ uptake experiment shown in **A**. The triangle on the right side of the blot serves as an indicator for the complex glycosylated band of TRPV5.

The p.(Val598Met) residue is located in the TRP helix region of TRPV5, which plays an instrumental role in controlling the channel pore gating, folding, and assembly. Initially, we visualized the wildtype and TRPV5 p.(Val598Met) variant structure models in UCSF Chimera, using an existing full-length wildtype TRPV5 structure and Dynamut2 prediction software [19–21]. We found that the TRPV5 p.(Val598Met) variant is more tightly contacted by surrounding structures (Fig. 3A, B). DynaMut2 prediction also suggests that the variant decreases protein flexibility. Interestingly, several papers have highlighted that the TRP channel lower pore opening and closing depends on movement within the S4-S5 linker, the TRP helix, and the pore helices [22, 23]. In addition, a new group of transient potential ankyrin type 1 (TRPA1) antagonists, a channel that is structurally related to TRPV5, have been shown to antagonize transitional movement in the S4-S5 linker and TRP helix, which are required for channel opening [24]. Taken together, these studies suggest that reduced flexibility in the TRP helix could have detrimental effects on protein function. As such, we investigated whether the channel function of the TRPV5 p.(Val598Met) variant is altered. To this end, TRPV5 wildtype and p.(Val598Met) plasmids were transfected in HEK293 cells and TRPV5-dependent Ca^2+^ transport was quantified using a ^45^Ca^2+^ uptake assay. Uptake is significantly increased in HEK293 cells that express the wildtype TRPV5 channel, compared to mock-transfected HEK293 cells (*p* = 0.0026) (Fig. 3C). In contrast, transfection with the TRPV5 p.(Val598Met) mutant channel produces ^45^Ca^2+^ uptake similar to mock-transfected cells (*p* = 0.6), suggesting that the TRPV5 p.(Val598Met) channel is non-functional. Functional TRPV5 channels consist of four subunits. To see whether TRPV5 p.(Val598Met) could have a dominant negative effect on the channel activity (simulating the heterozygous state), we co-transfected TRPV5 wildtype and p.(Val598Met) plasmids (1:1 ratio) in HEK293 cells. While there is a slight decrease in ^45^Ca^2+^ uptake compared to the TRPV5 wildtype condition, this is likely due to less *TRPV5* wildtype DNA used in the co-transfection setup than in the condition that contains only *TRPV5* wildtype DNA (Fig. 3C). In support of this, there is no significant difference (*p* = 0.2119) compared to the co-transfection of *TRPV5* and mock (condition added to control for DNA amount) (Fig. 3C), indicating that TRPV5 p.(Val598Met) does not produce a dominant negative effect.

We further investigated the underlying cause of the loss of function seen in the TRPV5 p.(Val598Met) channel by measuring protein expression via immunoblotting in TRPV5 wildtype and p.(Val598Met) transfected HEK293 cells. Interestingly, the TRPV5 p.(Val598Met) mutant channel lacks the complex-glycosylation band typically seen when visualizing TRPV5 (Fig. 3D). The condition where TRPV5 wildtype and TRPV5 p.(Val598Met) are co-transfected also showed reduced complex-glycosylation.

### TRPV5 p.(Val598Met) mutant channel plasma membrane insertion is unaffected but proteasomal degradation is enhanced

**Fig. 4:**
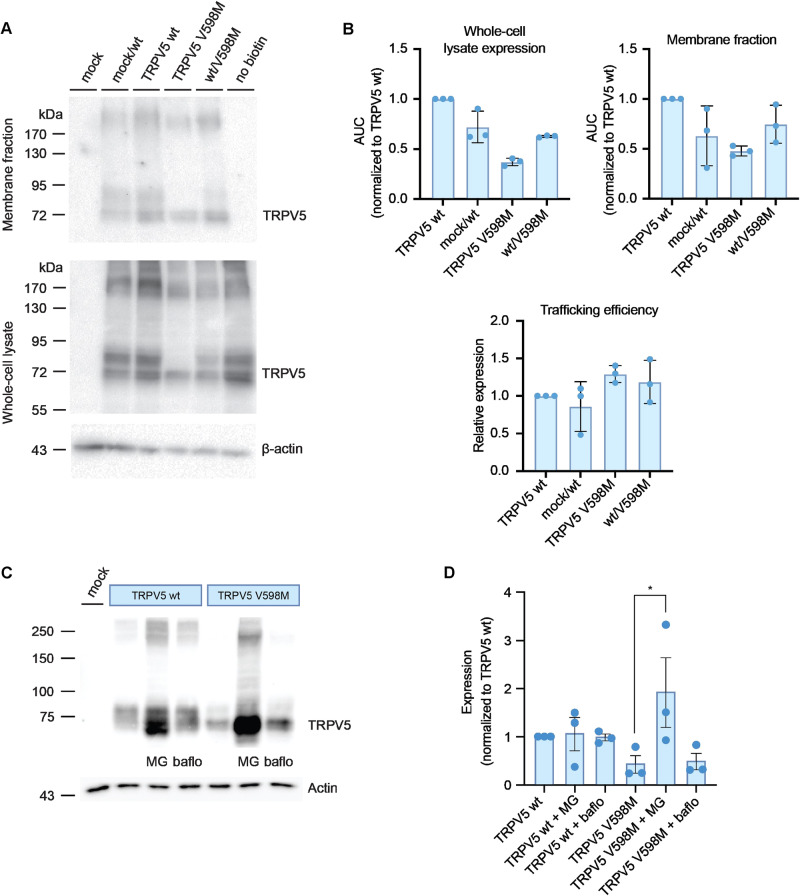
Trafficking efficiency of TRPV5 p.(Val598Met) is not altered, but proteasomal degradation is increased. **A** Representative immunoblot of a cell-surface biotinylation experiment in HEK293 cells transfected with TRPV5 wildtype (wt) and TRPV5 p.(Val598Met). The top panel shows the membrane fraction whereas the bottom panel corresponds to the whole-cell lysate fraction. No biotin control, where biotin is not added to the cells, confirms that there is no non-specific binding of unbiotinylated TRPV5 to the neutravidin beads. **B** Semi-quantification of expression signal of TRPV5 wt and TRPV5 p.(Val598Met) protein, plotted as the area under the curve (AUC), normalized to the TRPV5 wt expression. The top two panels show the whole-cell lysate and membrane fraction expression respectively, while the bottom panel depicts the trafficking efficiency as the ratio of membrane fraction expression divided by whole-cell lysate expression. The individual data points indicate the 3 independent biological replicates (*N* = 3). **C** Representative immunoblot of HEK293 cells transiently transfected with TRPV5 wt and TRPV5 p.(Val598Met) (TRPV5 V598M), treated with inhibitors of proteasomal and lysosomal protein degradation pathways. MG = MG-132 and baflo = Bafilomycin A1. **D** Semi-quantification of TRPV5 wt and TRPV5 p.(Val598Met) expression levels in 3 independent experiments where MG-132 and Bafilomycin A1 were added. Expression levels are normalized to the untreated TRPV5 wt condition. The asterisk denotes the significant difference between the conditions TRPV5 p.(Val598Met) without any inhibitors and TRPV5 p.(Val598Met) with Bafilomycin A1 (*p* < 0.05).

The lack of complex-glycosylation on the TRPV5 p.(Val598Met) channel could point to a trafficking or folding defect that prevents TRPV5 p.(Val598Met) from reaching the plasma membrane. As such, the abundance of both channels at the plasma membrane was assessed with a cell-surface biotinylation assay. The abundance of TRPV5 p.(Val598Met) is clearly diminished in both the plasma membrane and whole-cell fractions, compared to the wildtype channel (Fig. 4A). However, the plasma membrane to whole-cell TRPV5 expression ratio is similar between all of the conditions (Fig. 4B), indicating no trafficking defect of TRPV5 p.(Val598Met) to the plasma membrane. The fact that TRPV5 p.(Val598Met) expression levels are lower in both cellular compartments, suggests a more upstream effect. To test this further, we treated TRPV5 wildtype and p.(Val598Met) transfected HEK293 cells with inhibitors of lysosomal (bafilomycin A1) and proteasomal (MG-132) protein degradation. Interestingly, inhibition of the proteasomal degradation rescued non-glycosylated TRPV5 p.(Val598Met) protein expression levels, while inhibition of the lysosomal protein degradation had no effect (Fig. 4C, D). Neither inhibitor changed the expression level of the TRPV5 wildtype, indicating that the rescue observed in the TRPV5 p.(Val598Met) condition is due to targeting of this defective protein to the proteasome.

## Discussion

Renal Ca^2+^ reabsorption by DCT and CNT is an important factor in determining the blood Ca^2+^ level and is under strict endocrine control [8]. The apical membranes of cells lining these tubules harbor TRPV5 as the central protein controlling calcium permeability. Herein, using a combination of homozygosity mapping and WES, we describe the homozygous missense variant (c.1792G>A; p.(Val598Met)) in *TRPV5* that causes renal Ca^2+^-wasting hypercalciuria (RCWH). Functional characterization of the TRPV5 p.(Val598Met) mutant in HEK293 cells revealed that the channel is incapable of transporting significant amounts of Ca^2+^. TRPV5 p.(Val598Met) mutant had a complete absence of complex N-linked glycosylation and was also subject to increased proteasomal degradation.

Since its discovery in 1999, *TRPV5* has been in the spotlight as a candidate gene causing IH. Despite the expectation, extensive GWASs failed to demonstrate any association between common variants in *TRPV5* and nephrolithiasis/IH. However, in a large case-control study from Iceland, the rare missense *TRPV5* variant (rs757494578, c.1589T>G; p.(Leu530Arg)) was associated with recurrent kidney stones [14]. The Ca^2+^ transport defect and the absence of complex-glycosylation that were observed in the TRPV5 p.(Leu530Arg) variant are remarkably similar to our observations for the p.(Val598Met) mutant channel [25]. Each TRPV5 monomer is synthesized as a 6-pass transmembrane protein in the endoplasmic reticulum (ER), undergoes N-linked glycosylation at p.Asn358 which is completed as complex-glycosylation in Golgi apparatus, and finally reaches the plasma membrane [11, 26, 27]. While it is noteworthy that both of these variants lose their complex-glycosylation, it does not fully explain the loss of transport function. Studies have shown that disruption of TRPV5 complex-glycosylation, via a p.(Asn358Gln) mutation, does not drastically reduce ^45^Ca^2+^ uptake and trafficking to plasma membrane in HEK293 cells [28].

We initially speculated, based on the decreased flexibility predicted by structural modeling and the observed loss-of-function effect, that channel pore opening and closing may be affected in the TRPV5 p.(Val598Met) mutant. However, the follow-up experiments showed that the loss-of-function effect could be attributed to increased breakdown of the mutant channel and a potential folding defect. Furthermore, a loss of complex-glycosylation can point to a folding defect in the mutated protein. It is known that proteins that repeatedly fail their folding checkpoints in the ER are de-mannosylated and transported to the cytosol, where they are broken down by the proteasome [29]. A well-known example is the cystic fibrosis transmembrane conductance regulator (CFTR) channel. Studies have shown that mutated versions of CFTR lose their complex-glycosylation tree, fail to fold correctly, and are subsequently broken down [30]. To check if this also applies to TRPV5, we inhibited the two main pathways of protein degradation. Inhibition of the proteasomal degradation by MG132, which reduces the degradation of mutant CFTR, resulted in the rescue of TRPV5 p.(Val598Met) protein, showing that the mutant protein is recognized as misfolded and targeted for destruction.

Although a well-defined *TRPV5*-related phenotype has not been previously described in humans, the critical role of *Trpv5* in renal Ca^2+^ handling has long been demonstrated in mice [12, 31]. *Trpv5*-knockout mice (*Trpv5*−/−) present with marked hypercalciuria as a result of renal Ca^2+^ wasting, which is compensated by an increase of 1,25-dihydroxy vitamin D_3_ leading to normocalcemia. Additionally, these mice have PTH levels comparable to control mice [12]. This phenotypic picture is strikingly similar to the clinical phenotype observed in the individuals with homozygous *TRPV5* mutations presented here (II-1, II-2, II-6), except for hyperparathyroidism in the proband. On the other hand, the heterozygous mice (*Trpv5* + /−) do not have any significant hypercalciuria compared to wildtypes, which is in line with our observations that only show an insignificant reduction of TRPV5 function in double-transfected HEK293 cells [12]. These are in parallel with the phenotype of the heterozygotes (I-1, I-2, II-4, II-5) who either are not hypercalciuric or exhibit non-persistent mild hypercalciuria (Supplementary Table S1). It is known that dietary factors have a significant impact on calcium excretion [32]. Thus, it is likely that the non-persistent hypercalciuria in the unaffected family members is due to temporary environmental factors. Even so, any possible contribution of pathogenic heterozygous *TRPV5* variants to urinary calcium excretion in humans is yet to be explored.

The severe skeletal findings and unexpectedly high PTH levels which are neither observed in the proband’s siblings nor the *Trpv5*-knockout mice are highly indicative of an additional intrinsic bone disorder in individual II-2. In fact, the mice lacking Trpv5 show no major bone deformation even at long-term follow-up (up to 78 weeks) [33, 34]. However, these mice demonstrate an accelerated reduction in trabecular and cortical bone thickness [12, 35]. Similar to the proband presented here, knockout mice do not manifest any increase in the bone resorption marker urinary deoxypyridinoline, while the bone formation marker, serum osteocalcin, is significantly higher compared to wild-type mice. In contrast to observations in the knockout mice, another bone formation marker, ALP, is strikingly higher in the proband, which also differs from his siblings with homozygous p.(Val598Met) [12].

All in all, the proband’s severe bone phenotype accompanied by increased PTH and ALP cannot be explained only by the *TRPV5* defect. In recent years, with the widespread diagnostic utilization of WES, previously underestimated blended phenotypes have frequently been discovered, reaching up to 7.5% in certain populations [36]. Based on this observation, the search for an additional variant in the proband using a virtual hypercalciuria/hyperparathyroidism panel for disease-causing variants did not uncover any other germline variant (Supplementary Table S2). Nevertheless, a search for somatic variants revealed the mosaic pathogenic *GNAS* variant (p.(Arg201Cys)) which is a well-known cause of fibrous dysplasia. Considering the involvement of multiple long bones, increased serum ALP levels, and absence of *GNAS*-related McCune-Albright-Syndrome-associated extraskeletal findings, the skeletal deformities seen in the proband are compatible with POFD [37, 38]. Clinical presentation in POFD becomes apparent during early childhood and progresses until adulthood despite normal in-utero skeletal development similar to the clinical course seen in the proband [38]. However, hypercalciuria is inexplicable solely by fibrous dysplasia, because hypercalciuria has only been mentioned in a few cases of fibrous dysplasia that are complicated by Cushing’s Syndrome [39, 40]. Thus, the hypercalciuria is a consequence of the *TRPV5* mutation, but not the *GNAS* mutation, in the proband.

Hyperparathyroidism in the proband can be explained neither by *GNAS* nor *TRPV5* mutations alone. However, it is possible that hyperparathyroidism may arise as a result of an additive effect of these mutations [36]. Some individuals with fibrous dysplasia can have hyperparathyroidism due to accompanying vitamin D deficiency or primary hyperparathyroidism (parathyroid adenoma, hyperplasia), which are both ruled out in the proband [41–44]. The observation that elder *Trpv5*^*−/−*^ mice demonstrate severely elevated serum PTH in contrast to young knockout mice may provide some explanation for the hyperparathyroidism seen in the proband [45]. It has been proposed that the elevated PTH level is a necessary compensatory mechanism to sustain normocalcemia in elder *Trpv5*^*−/−*^ mice that cannot tolerate the age-related changes in intestinal and bone Ca^2+^ metabolism due to the already strained Ca^2+^ homeostasis [45]. The POFD in the proband undoubtedly burdens the Ca^2+^ metabolism and decreases the bone mineral density, which can presumably be compensated by increased PTH [42]. Normal PTH levels in proband’s affected siblings without the somatic *GNAS* mutation support the additive effect hypothesis. However, it is yet to be seen if hyperparathyroidism would arise in these siblings with advancing age.

Blended phenotypes caused by a combination of germline and somatic variants are quite rare and primarily observed in hematological disorders that involve a germline disorder ameliorated by indirect somatic genetic rescue [46, 47]. A previous study showed that osteoclast activity is decreased in *Trpv5*^*−/−*^ mice, whereas fibrous dysplasia lesions demonstrate excessive osteoclast activation due to increased Gsα signaling [35, 48, 49]. Considering the contrasting impact of *TRPV5* and *GNAS* mutations on osteoclastic activity, it is reasonable to postulate that the acquisition of a somatic *GNAS* mutation would lead to a transient and partial rescue of defective osteoclastic activity due to *TRPV5* mutation.

In conclusion, comprehensive genetic and functional studies performed here demonstrate that *TRPV5* is involved in autosomal recessively inherited IH through increased renal calcium excretion, which we name “Renal Calcium-Wasting Hypercalciuria” (RCWH). The anticipated relation between *TRPV5* and IH is now demonstrated in humans after >20 years. The complete loss of function seen in the TRPV5 p.(Val598Met) channel, coupled with the unexpected autosomal recessive inheritance pattern suggests that a complete absence of TRPV5 function may be required to cause RCWH in childhood. The recessive inheritance pattern and extremely low allele frequency of the *TRPV5* variant described in this work suggest that RCWH could be found most often in highly consanguineous populations and emphasize the importance of exploration of this disorder within those communities.

## Supplementary information

Supplementary Method Tables and Figures

Supplementary Table S1

## Data Availability

Disease-causing *TRPV5* variants have been submitted to ClinVar with the accession number SCV003852754. All data for cell culture studies were collected and saved in accordance with the FAIR principles. The whole-exome sequencing, SNP microarray, and other raw data are available upon reasonable request.
