# Supplementary material for: Decreased calcium permeability caused by biallelic TRPV5 mutation leads to autosomal recessive renal calcium-wasting hypercalciuria
Source: Eur J Hum Genet. 2024 Mar 25;32(11):1506–14. doi: 10.1038/s41431-024-01589-9 (PMC11577068; doi:10.1038/s41431-024-01589-9)
Supplement: Supplementary file 1 — Supplementary Method Tables and Figures [file 41431_2024_1589_MOESM1_ESM.pdf]

SUPPLEMENTARY METHODS

DNA Sequencing

Sequence reads were aligned to hg19/GRCh37 human reference genome using BWA (1). Duplicated reads were marked using Picard v2.25.5 and variants were called following best practices for HaplotypeCaller and Mutect2 tools from Genome Analysis Toolkit 4.2 for germline and somatic variants, respectively (2-4). Germline variants that covered exonic regions and 20 bp around exon boundaries were called using a .bed file created from hg19 UCSC genes (5). Called variants were hard filtered using bcftools v1.13 and vcftools v0.1.13 and annotated by ANNOVAR v20200608 (6-8). Annotated .vcf files were filtered following various strategies to single out monogenic disease variants.

The presence of multiple individuals with hypercalciuria and consanguinity between parents strongly suggests autosomal recessive inheritance. Based on this, we first focused on germline variants within shared homozygosity mapping regions. Shared homozygosity blocks in either all individuals with hypercalciuria (II:1, II:2, II:6) or long contiguous stretches of homozygosity (LCSHs) unique to the proband (II:2) were laid out using homozygosity mapping. Furthermore, due to the additional skeletal phenotype in the proband, germline and somatic variants irrespective of zygosity from WES data were filtered using a "Virtual Skeletal Disorders panel" that contains a total of 563 genes either listed in the 10th version of the nosology of genetic skeletal disorders (9), included among the genes attributed to Paget disease of bone in OMIM (10), or the genes reviewed as having high or moderate evidence at least once in the "Skeletal Dysplasia Panel" or "Osteogenesis Imperfecta Panel" in the Genomics England PanelApp repository (11,12). Finally, another panel containing 46 genes, which we name "Virtual Hypercalciuria/Hyperparathyroidism panel" was constructed from recent reviews in the literature and high or moderate evidence genes within the Genomics England PanelApp repository for "Familial Hyperparathyroidism or Hypocalciuric Hypercalcaemia" or "Familial Hyporparathyroidism" (13-17). in the Germline variants with Genotype Phred scores (GQ) ≥30, read depth ≥20x, somatic variants with read depth ≥50x, and allele frequency between 0.1 and 0.4 were considered high-quality and suitable for further analysis. Among these variants, those with low Minor Allele Frequency (MAF<0.01) either in gnomAD, ExAC, the 1,000 Genomes projects, or the in-house database of clinical next-generation sequencing data, that are seen in homozygous/hemizygous state for no individuals or in heterozygous state for ≤3 individuals in gnomAD, and within exons or intronic regions ≤8 bp distance to the closest intron-exon boundary were filtered in (18). These variant lists were further filtered for those that are synonymous, residing >3 bp distance to the closest intron-exon boundary, and those with genotypes contradicting the reported inheritance pattern for the gene to build a final list of candidate genes.

RNA Studies

Total peripheral whole blood RNA from the proband (II-2) was extracted using the QIAamp RNA Blood Mini Kit (Qiagen, Hilden, Germany). Complementary DNA (cDNA) was reverse-transcribed (RT) from total RNA using ipsogen RT Kit (Qiagen, Hilden, Germany) with random 9 bp primers, following the manufacturer's protocol. Specific primer pairs flanking exons on either side of the candidate *POP1* variant (NM_001145860.1:c.489G>A, p.Ala163Ala) were used for polymerase chain reactions (PCR) of respective cDNA sequences. PCR products were

visualized by 2% (w/v) agarose gel electrophoresis and sequenced by the dideoxy chain termination method as described above.

Generation of TRPV5 p.Val598Met Construct

In short, mutagenesis PCR was used on a pCINEO-IRES GFP construct that contained the HA-tagged wildtype *TRPV5* open-reading frame (ORF), using specific primers to induce the p.Val598Met mutation. Products were incubated with Q5 KLD mix for 5 minutes at room temperature and transformed into NEB 5-alpha competent *E.Coli* bacteria with standard heat-cold shock transformation. DNA was isolated from the cells with a Nucleobond mini-prep kit (Macherey-Nagel, Dueren, Germany), according to the manufacturer's protocol. The resulting *TRPV5* p.Val598Met mini-prep was re-transformed in competent Top10F *E.Coli* and DNA was isolated using the Nucleobond midi-prep kit (Macherey-Nagel, Dueren, Germany). The resulting *TRPV5* p.Val598Met ORF, CMV promoter, and GFP tag were sequenced using Sanger sequencing.

Cell Culture and Transfection

HEK293 cells were purchased from ATCC (LGC Standards GmbH, Wesel, Germany) and cultured in Dulbecco's Modified Eagle's Medium (DMEM, Gibco, Life Technologies Europe BV, Netherlands), supplemented with 10% (v/v) fetal bovine serum, 2 mM L-glutamine and 10 µg/ml non-essential amino acids. Cells were incubated at 37°C in a humidity-controlled incubator with 5% (v/v) CO_2. Cells were transfected with 2 µg of DNA using Lipofectamine2000 (Thermo Fisher Scientific, Waltham, MA, USA) as a transfection reagent, at a DNA:Lipofectamine2000 ratio of 1:2, according to the manufacturer's protocol. In the experiments that assessed the effect of the mutation on protein stability, inhibitors of proteasomal (MG-132 (MG), 5 µM, obtained from Merck, Darmstadt, Germany) and lysosomal (Bafilomycin A1 (Baflo), 100 nM, obtained from Cell Signalling Technology, Beverly, MA, USA) degradation were added 14 hours prior to preparation of protein lysates for SDS-PAGE and Western blotting.

Biotinylation

HEK293 cells were seeded onto poly-L-lysine-coated 6-well plates and transfected approximately 6 hours later. 48 hours post-transfection, cells were transferred to a cold room where they were washed three times with ice-cold PBS-CM (PBS, 1 mM $MgCl_2$, 0.5 mM $CaCl_2$ at a pH of 8.0, set with NaOH) before being incubated with 0.5 mg/ml biotin (EZ-link Sulfo-NHS-LC-LC biotin, Thermo Fisher Scientific, Waltham, MA, USA) for 30 minutes at 4°C under gentle agitation. The biotinylation binding reaction was stopped by washing the cells twice with PBS-BSA (PBS with 0.1% (w/v) bovine serum albumin) and once with PBS alone. After quenching, the cells were lysed in 0.5 ml lysis buffer (50 mM Tris-HCl (pH 7.5), 150 mM NaCl, 1 mM EDTA, 1 mM EGTA, 1% (v/v) Triton X-100, 1 mM sodium orthovanadate, 10 mM sodium-glycerophosphate, 50 mM sodium fluoride, 10 mM sodium pyrophosphate, 270 mM sucrose, and the freshly added protease inhibitors pepstatin A (1 µg/ml), PMSF (1 mM), leupeptin (5 µg/ml), and aprotinin (1 µg/ml)) for 1 hour on a shaking platform. Afterwards, cell lysates were collected in 1.5 ml tubes and centrifuged for 15 min at 16,000xg to get rid of detergent-insoluble material. The supernatant was transferred to new 1.5 ml tubes and protein concentration was determined using the Bradford method. Equal amounts of protein (1250 µg) were loaded onto 40 µl neutravidin bead

slurry (Pierce biotechnology, 50:50 ratio of neutravidin beads with PBS). Bead samples were supplemented with lysis buffer (without protease inhibitors) to a total volume of 1 ml and rotated overnight at 4°C. Beads were spun down at 2,000xg for 2 minutes and washed with fresh lysis buffer. This step was repeated two more times to remove all unbound biotin-tagged proteins. After the third wash, all lysis buffer was removed from the beads and 40 µl of 2x SDS sample buffer (4% (w/v) SDS, 10% (v/v) β-mercaptoethanol, 20% (v/v) glycerol, 120 mM Tris-HCl (pH 6.8), and 0.02% (v/v) bromophenol blue) was added. Samples were then incubated for 10 minutes at 70°C.

SDS-PAGE and Western Blotting

Samples were separated on 8% (w/v) SDS-PAGE gels with electrophoresis equipment and transferred onto PVDF membranes with standard Western blotting technique. The membranes were blocked for 30 minutes at room temperature with 5% (w/v) non-fat dry milk in TBS-T (TBS with 2% (v/v) Tween20) and incubated overnight on a rocker system with primary antibodies against the HA-tag on TRPV5 (1:5,000, Cell Signalling Technology) or anti-beta-Actin (1:10,000, Sigma A5441, Merck, Darmstadt, Germany) as the loading control. Blots were washed in TBS-T, in four wash steps of 15 minutes, to remove unbound primary antibodies. Afterwards, blots were incubated with horseradish peroxidase-coupled (HRP-coupled) secondary goat-anti-mouse IgG antibody (1:10,000, Chemie Brunschwig, Basel, Switzerland) for 1 hour at room temperature on a rocker system. Blots were developed with Pierce ECL western blotting substrate and visualized in a Bio-Rad Chemidoc XRS setup. Analysis of the blots was done in the Fiji suite of ImageJ (19).

Radioactive $^{45}Ca^{2+}$ Uptake

HEK293 cells were seeded on 6-well plates (1 well per condition) and transfected 4-6 hours later with the appropriate constructs. 24 hours later, cells were detached with trypsin and replated as technical triplicates into poly-L-lysine-coated (0.1 mg/ml) 24-well plates. Radioactive $^{45}Ca^{2+}$ uptake was performed according to a previously described protocol (20). In short, HEK293 cells in 24-well plates were pre-treated at 37°C with the cell-permeant calcium chelator BAPTA-AM (25 µM, Thermo Fischer Scientific), dissolved in Opti-MEM (Thermo Fischer Scientific), for 30 minutes prior to the experiment. Cells were subsequently washed with KHB buffer (which contains 10 mM NaCl, 5 mM KCl, 1.2 mM $MgCl_2$, 0.1 mM $CaCl_2$, 10 mM Na-acetate, 2 mM NaH_2PO_4, and 20 mM HEPES, set to a pH of 7.4 with NaOH) and incubated for 10 minutes at 37°C with $^{45}Ca^{2+}$ (±1µCi/ml) in KHB buffer supplemented with the voltage-gated Ca^{2+} channel blockers felodipine and verapamil (both at 10 µM concentration). The TRPV5 blocker ruthenium red (10 µM) was used to gauge the amount of TRPV5-dependent $^{45}Ca^{2+}$ uptake. After the 10-minute incubation step, cells were washed thrice in ice-cold stop buffer (which contains 110 mM NaCl, 5 mM KCl, 1.2 mM $MgCl_2$, 0.5 mM $CaCl_2$, 10 mM Na-acetate, 1.5 mM $LaCl_3$, and 20 mM HEPES, set to a pH of 7.4 with NaOH) and lysed in 0.05% (w/v) SDS for 30 minutes at 37°C. Lysates were transferred to tubes containing 4 ml Opti-Fluor scintillation fluid (Perkin Elmer, Waltham, WA), vortexed briefly, and the amount of $^{45}Ca^{2+}$ uptake was measured using a scintillation counter.

Statistical Analysis

Data points for semi-quantified band densities from Western Blots and scintillation counts for radioactive $^{45}Ca^{2+}$ uptake assay were transferred and analyzed in Graphpad Prism 9.0

(GraphPad Software Inc.). Data are shown as averaged bar graphs ± standard error of the mean (SEM), also denoting the independent experimental data points. The statistical method that was used to assess differences between the conditions is a Kruskal-Wallis test in conjunction with Dunn’s multiple comparisons test, considering $p < 0.05$ as statistically significant.

SUPPLEMENTARY TABLE

Supp. Table S2: Stepwise filtering of WES variants in the proband.

Variants	LCSH regions unique to proband	LCSH regions in 3 affected individuals	Skeletal Disorders		Virtual Hyper-calciuria/ Hyper-PTH Panel		Somatic Variants
**Total**	51,791	51,791	51,791		51,791		41,805
**Candidate regions**	301 (within LCSH regions)	544 (within LCSH regions)	1445 (within Virtual Skeletal Disorders Panel)		110 (within Virtual Hypercalciuria/ Hyper-PTH Panel)		61 (0.1≤VAF ≤0.4 and within Virtual Skeletal Disorders Panel)
**GQ≥30 and read depth ≥20**	240	361	1,135		87		NA
**Zygosity**	181 (hom)	268 (hom)	471 (hom)	664 (het)	38 (hom)	49 (het)	NA
**MAF≤0.01 in ExAC, gnomAD, TGP**	7	6	4	48	1	3	7
**Not observed in homozygous state in gnomAD v4.0**	3	3	3	24[*]	1	1[*]	6
**Missense and predicted loss of function, validated by IGV inspection**	2	3	3	15	1	0	2
**Compatible with OMIM inheritance pattern or inheritance unknown**	2	3	3	7	1	0	1 (not germline)
**Genes containing candidate variant**	<u>*POP1*</u>[‡]<br>*TSPAN6*[***]	*MROH9*[***]∅<br><u>*METTL11B*</u>[†]∅<br>***TRPV5*** ↑	*CSPP1*[**]<br><u>*POP1*</u>[‡]<br><u>*IDS*</u>[**]	*TRPV4*[**]<br>*NIN*[**]<br>*ANKRD11*[@]<br><u>*RPL13*</u>[**]<br>*CFAP410*[**]	***TRPV5***	-	***GNAS***

ExAC, The Exome Aggregation Consortium. gnomAD, The Genome Aggregation Database. Het, Heterozygous. Hom, Homozygous. LCSH, Long Contiguous Stretches of Homozygosity. MAF, Minor Allele Frequency. NA, Not Applicable. OMIM, Online Mendelian Inheritance in Man. TGP, The Thousand Genomes Project. VAF, Variant Allele Frequency. [*]These heterozygous variants are also not present in ≥3 individuals in gnomAD. [**] eliminated due to non-segregation. *** eliminated due to the presence of homo/hemizygous individuals with other predicted loss of function variants in gnomAD. [‡] eliminated due to no effect on splicing (see Supplementary Fig. S1). [†] eliminated due to no significant phenotypes in reported mouse models. [@] eliminated due to high frequency in the in-house database. The GTEx expression levels of indicated genes in kidney are either none (∅) or high (↑) (21). The underlined genes indicate variants that are close to a splice site, while others are missense/nonsense variants.

SUPPLEMENTARY FIGURES

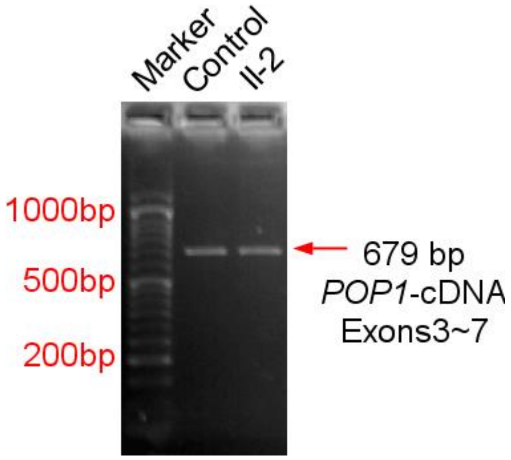

Supplementary Fig. 1: The effect of *POP1*:c.489G>A variant on splicing. Agarose gel electrophoresis shows that splicing is not affected by a candidate variant near the exon-intron boundary (*POP1*:c.489G>A) compared to the control.

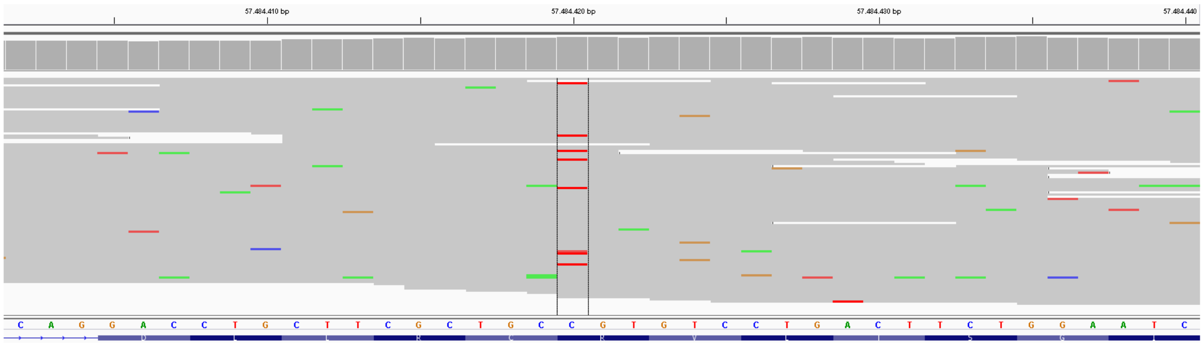

Supplementary Fig. 2: Visualization of the mosaic *GNAS*:c.601C>T variant from WES data. Visualization of the aligned reads by Integrated Genomics Viewer shows that *GNAS*:c.601C>T variant is present only in ~10% of the reads overlapping this position, confirming the mosaicism.

SUPPLEMENTARY REFERENCES:

1- Li H, Durbin R. Fast and accurate long-read alignment with Burrows–Wheeler transform. Bioinform. 2010;26(5):589-595.
2- Benjamin D, Sato T, Cibulskis K, Getz G, Stewart C, Lichtenstein L. Calling somatic SNVs and indels with Mutect2. BioRxiv. 2019:861054.
3- Picard [Internet]. Picard Tools version 2.17.8-By Broad Institute. [cited 2018 Feb 21]. Available from: https://broadinstitute.github.io/picard/.
4- Van der Auwera GA, O'Connor BD. Genomics in the cloud: using Docker, GATK, and WDL in Terra: O'Reilly Media; 2020.
5- UCSC Table Browser [Internet]. [cited 2023 Mar 15]. Available from: http://genome.ucsc.edu/cgi-bin/hgTables?hgsid=1597875591_Xgd6ICemLeYyWix5GdjBmM8xECL4&clade=mammal&org=Human&db=hg19&hgta_group=genes&hgta_track=knownGene&hgta_table=0&hgta_regionType=genome&position=chr2%3A25%2C383%2C722-25%2C391%2C559&hgta_outputType=bed&hgta_outFileName=.
6- Danecek P, Auton A, Abecasis G, Albers CA, Banks E, DePristo MA, et al. The variant call format and VCFtools. Bioinform. 2011;27(15):2156-2158.
7- Li H. A statistical framework for SNP calling, mutation discovery, association mapping and population genetical parameter estimation from sequencing data. Bioinform. 2011;27(21):2987-2993.
8- Wang K, Li M, Hakonarson H. ANNOVAR: functional annotation of genetic variants from high-throughput sequencing data. Nucleic Acids Res. 2010;38(16):e164.
9- Mortier GR, Cohn DH, Cormier-Daire V, Hall C, Krakow D, Mundlos S, et al. Nosology and classification of genetic skeletal disorders: 2019 revision. Am J Med Genet. A. 2019;179(12):2393-2419.
10- Phenotypic Series-Paget Disease of Bone-OMIM [Internet]. [cited 2023 Mar 15]. Available from: https://www.omim.org/phenotypicSeries/PS167250.
11- Skeletal Dysplasia Panel [Internet]. [cited 2022 May 15]. Available from: https://panelapp.genomicsengland.co.uk/panels/309.
12- Osteogenesis Imperfecta Panel [Internet]. [cited 2022 May 15]. Available from: https://panelapp.genomicsengland.co.uk/panels/196.
13- Downie ML, Alexander RT. Molecular mechanisms altering tubular calcium reabsorption. Pediatr Nephrol. 2022:1-12.
14- Howles SA, Thakker RV. Genetics of kidney stone disease. Nat Rev Urol. 2020 Jul;17(7):407-421.
15- Singh P, Harris PC, Sas DJ, Lieske JC. The genetics of kidney stone disease and nephrocalcinosis. Nat Rev Nephrol. 2022 Apr;18(4):224-240.
16- Familial hyperparathyroidism or hypocalciuric hypercalcaemia Panel [Internet]. [cited 2023 December 28]. Available from: https://panelapp.genomicsengland.co.uk/panels/480.
17- Familial hypoparathyroidism Panel [Internet]. [cited 2023 December 28]. Available from: https://panelapp.genomicsengland.co.uk/panels/312.

18- Karczewski KJ, Francioli LC, Tiao G, Cummings BB, Alföldi J, Wang Q, et al. The mutational constraint spectrum quantified from variation in 141,456 humans. Nature. 2020;581(7809):434-443.
19- Schindelin J, Arganda-Carreras I, Frise E, Kaynig V, Longair M, Pietzsch T, et al. Fiji: an open-source platform for biological-image analysis. Nat Methods. 2012;9(7):676-682.
20- de Groot T, Lee K, Langeslag M, Xi Q, Jalink K, Bindels RJ, et al. Parathyroid hormone activates TRPV5 via PKA-dependent phosphorylation. J Am Soc Nephrol. 2009;20(8):1693-1704.
21- Lonsdale J, Thomas J, Salvatore M, Phillips R, Lo E, Shad S, et al. The genotype-tissue expression (GTEx) project. Nat Genet. 2013;45(6):580-585.
